# Acute Kidney Injury and Nephrotic‐Range Proteinuria as Initial Presentation of Pheochromocytoma: A Case Report

**DOI:** 10.1155/crie/8510972

**Published:** 2026-04-26

**Authors:** Anum Rizwan, Sajid Islam Bhatti, Huda Raja, Sidra German, Tajammul Waqar, Abdullah Nadeem, Nahid Raufi

**Affiliations:** ^1^ Department of Nephrology, Sindh Institute of Urology and Transplantation (SIUT), Karachi, Pakistan, siut.org; ^2^ Department of Medicine, Dow University of Health Sciences, Karachi, Pakistan, duhs.edu.pk; ^3^ Department of Medicine, Kabul Medical University, Kabul, Afghanistan

**Keywords:** acute kidney injury, adrenalectomy, catecholamine-secreting tumor, hypertension, nephrotic-range proteinuria, pheochromocytoma, renal dysfunction

## Abstract

**Introduction:**

Pheochromocytomas are rare catecholamine‐producing neuroendocrine tumors that present with the classical triad of paroxysmal hypertension, palpitations, and sweating; however, this presentation is seen in less than 1/4 of pheochromocytoma patients. Hypertension is usually the predominant manifestation. Atypical manifestations, including renal involvement, are infrequent and may delay diagnosis.

**Case Presentation:**

We report the case of a 37‐year‐old male with no prior comorbidities who presented with progressive abdominal pain, shortness of breath, and oliguria. Laboratory evaluation demonstrated acute kidney injury with nephrotic‐range proteinuria, initially raising suspicion of a primary glomerular disorder. Chest imaging revealed pulmonary congestion, while abdominal imaging identified a right adrenal mass. Plasma‐free metanephrine and normetanephrine levels were markedly elevated, confirming pheochromocytoma. The patient underwent preoperative optimization with alpha‐adrenergic blockade and supportive measures, including temporary hemodialysis. A right adrenalectomy was subsequently performed, leading to complete resolution of renal dysfunction and normalization of blood pressure.

**Conclusion:**

This case emphasizes the diagnostic challenge of pheochromocytoma when presenting with renal complications. Clinicians should consider adrenal tumors in the differential diagnosis of patients with unexplained acute kidney injury, as timely recognition and surgical intervention can result in full recovery.

## 1. Introduction

Pheochromocytoma is an uncommon catecholamine‐secreting tumor that arises from the chromaffin cells of the adrenal medulla, while similar extra‐adrenal tumors are termed paragangliomas [1]. The reported incidence is around 0.8 cases per 100,000 person‐years [2], although the true prevalence may be higher, as nearly half of the cases in one series were identified only at autopsy [3]. These tumors are typically diagnosed in the fourth or fifth decade of life, with no gender predilection [4]. Biochemically, most pheochromocytomas secrete epinephrine and norepinephrine, whereas ~23% of paragangliomas arising from parasympathetic tissue produce dopamine alone [5]. Clinically, hypertension is the predominant manifestation, as shown in an audit from two large African academic hospitals, but patients may also present with nonspecific features such as headache, palpitations, sweating, or anxiety, leading to their description as “the great mimic” [6]. However, the triad is only usually seen in less than 1/4 of pheochromocytoma patients.

Despite this, pheochromocytomas may occasionally present with atypical features that obscure the diagnosis. In particular, renal involvement is rare and not commonly described as an initial presentation. We report the case of a 37‐year‐old man with no prior comorbidities who presented with progressive abdominal pain, dyspnea, oliguria, and nephrotic‐range proteinuria, mimicking primary renal disease. However, further imaging revealed a large right adrenal mass.

This case highlights a highly unusual presentation of pheochromocytoma with renal dysfunction and nephrotic‐range proteinuria, underscoring the need to consider catecholamine‐secreting tumors in patients with unexplained renal abnormalities.

## 2. Case Presentation

A 37‐year‐old male, married with three children, resident of Korangi, and a professional driver, with no prior comorbidities, presented with a 6‐month history of diffuse abdominal pain, more pronounced in the right upper quadrant. Pain was mild, gradual in onset, partially relieved by paracetamol, and unassociated with nausea, vomiting, jaundice, or bowel changes.

He initially sought medical attention at multiple hospitals and received proton pump inhibitors and antispasmodics. During evaluation, serum creatinine was found to be elevated, prompting nephrology follow‐up.

He presented to the SIUT Hospital emergency on 22 November 2023 with worsening abdominal pain, shortness of breath, and reduced urine output over 1 week. Dyspnea progressed from exertion to rest, with orthopnea and paroxysmal nocturnal dyspnea. Peripheral edema, frothy urine, and progressive oliguria were noted. He had no history of hematuria, rash, photosensitivity, hemoptysis, visual, or auditory disturbances. Past medical, surgical, and family histories were unremarkable. He reported a 10‐year history of ghutka use.

On physical examination, the patient appeared alert but dyspneic. Jugular venous pressure was elevated, and significant pedal edema was observed up to the shins, suggesting fluid overload and possible cardiac involvement secondary to renal dysfunction. Chest auscultation revealed bilateral fine crepitations up to the mid‐zone posteriorly, consistent with pulmonary congestion. Cardiac examination demonstrated normal first and second heart sounds along with a third heart sound, indicating volume overload. Abdominal examination revealed severe tenderness in the right lumbar region without palpable organomegaly, while the central nervous system was grossly intact, showing no focal deficits.

Laboratory evaluation revealed significant renal impairment with elevated urea and creatinine, alongside hyponatremia, hyperkalemia, and metabolic acidosis, reflecting acute kidney injury. Hematological investigations showed anemia and mild thrombocytopenia, likely secondary to chronic illness or uremia. Liver function tests were markedly deranged, with significantly elevated transaminases and bilirubin, suggesting concomitant hepatic involvement, possibly due to hypoperfusion or drug exposure. Urinalysis demonstrated proteinuria of 3+ with numerous red and white blood cells, and the spot urine protein‐to‐creatinine ratio was markedly elevated at 20.45 mg/mg, indicative of nephrotic‐range proteinuria. Urine culture, however, was sterile, ruling out active infection.

Given the persistent proteinuria and acute kidney injury in the context of normal‐sized kidneys on imaging, autoimmune serology was performed. Antinuclear antibody testing returned positive, and complement levels showed reduced C3 with borderline C4, suggesting possible immune‐mediated glomerular involvement. However, ANCA and anti‐GBM antibodies were negative, making vasculitic or anti‐GBM disease less likely. A renal biopsy was performed, which revealed minor changes in glomeruli on light microscopy and moderate acute tubular injury with dystrophic calcification in tubular lumina. Immunofluorescence was negative, confirming that the glomerular involvement was minimal and the primary pathology was not autoimmune glomerulonephritis. This discrepancy led to further investigations.

Ultrasound of the whole abdomen revealed a large heterogeneous lesion in the right suprarenal region measuring 6.3 cm × 7.9 cm, abutting the kidney and liver, raising suspicion for a neoplastic process. Further imaging with a triphasic CT scan demonstrated a heterogeneously enhancing mass in the right adrenal gland with lateral wall breach, suggestive of an adrenocortical tumor. To confirm a functional adrenal tumor, plasma metanephrine and normetanephrine levels were measured and found to be markedly elevated at 3344.2 pg/mL and 16,391.2 pg/mL, respectively, confirming a diagnosis of pheochromocytoma.

During hospitalization, the patient required hemodialysis with ultrafiltration to manage fluid overload and uremia, resulting in symptomatic improvement and stabilization of oxygen saturation. Remarkably, renal function began to recover spontaneously, allowing discontinuation of dialysis. Once medically optimized, the patient underwent a right adrenalectomy. Postoperatively, renal function normalized, proteinuria resolved, and the patient remained dialysis‐independent. Histopathological examination of the excised adrenal mass confirmed pheochromocytoma. The patient was followed twice monthly during the first 6 months after restoration of renal function. Up to August 2025, there was no recurrence or deterioration of renal function, and follow‐up imaging showed no residual or recurrent adrenal lesion.

Given the presence of nephrotic‐range proteinuria, hematuria, and acute kidney injury with preserved renal size, an autoimmune glomerulonephritis was initially considered. This suspicion was supported by a positive antinuclear antibody and reduced complement levels. However, extended autoimmune serology, including ANCA and antiglomerular basement membrane antibodies, was negative, reducing the likelihood of vasculitic or immune‐complex–mediated disease. Importantly, renal biopsy demonstrated only minor glomerular changes with no crescents, immune deposits, or vasculopathy on immunofluorescence, while showing predominant acute tubular injury with dystrophic calcification. These findings were inconsistent with primary autoimmune glomerulonephritis and instead suggested secondary ischemic or toxic tubular injury. In conjunction with imaging evidence of a large adrenal mass and markedly elevated plasma metanephrine and normetanephrine levels, the diagnostic focus shifted toward pheochromocytoma as the underlying cause of the renal and systemic manifestations.

**Figure 1:**
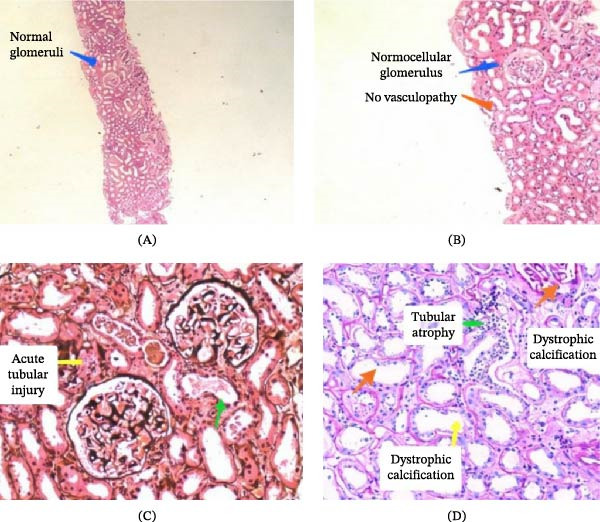
(A–D) Renal histopathology showing minor glomerular changes, moderate acute tubular injury with dystrophic calcification, minimal focal tubular atrophy, and negative immunofluorescence.

Figure 1A–D shows glomeruli that showed minor changes. No crescents, no vasculopathy. Moderate acute tubular injury is seen, along with dystrophic calcification in the tubular lumen. Minimal focal tubular atrophy seen. IMF was completely negative on paraffin tissue.

**Figure 2:**
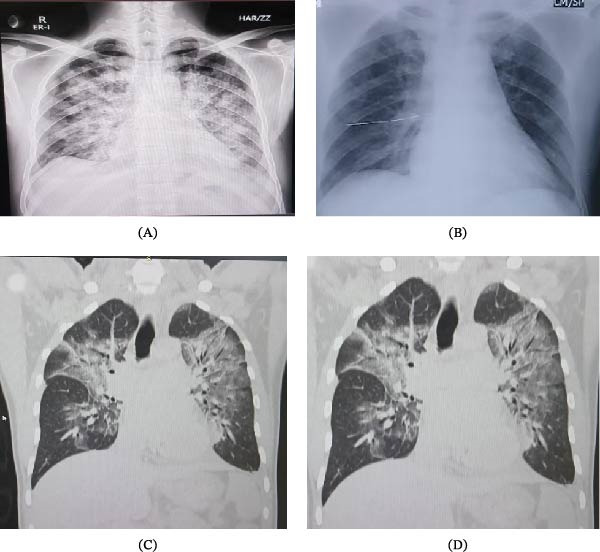
(A–D) Imaging findings of pulmonary congestion/edema. (A) Initial chest X‐ray demonstrating bilateral patchy perihilar and basal opacities, consistent with pulmonary congestion or edema. (B) Follow‐up chest X‐ray showing significant resolution of prior pulmonary congestion after intervention. (C, D) HRCT chest images showing diffuse ground‐glass opacities with interlobular septal thickening in bilateral lungs, consistent with pulmonary edema; no significant consolidation or cavitation is noted.

Initial imaging (Figure 2A) revealed bilateral patchy perihilar and basal opacities consistent with pulmonary congestion, correlating with the patient’s dyspnea, orthopnea, and renal dysfunction. Follow‐up chest X‐ray (Figure 2B) demonstrated marked improvement in lung fields post‐intervention. HRCT images (Figure 2C, D) confirmed diffuse ground‐glass opacities with septal thickening, supporting pulmonary edema secondary to volume overload, with no features suggestive of infection.

**Figure 3:**
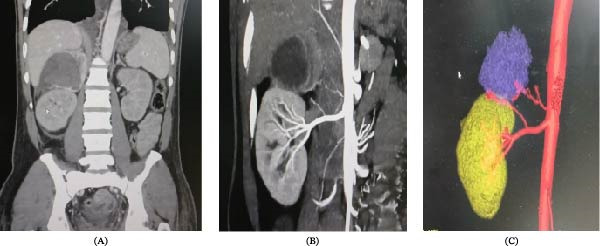
(A) Abdominal CT (adrenal region), coronal abdominal CT: A heterogeneous right suprarenal mass is visible, abutting the kidney and liver. (B) Contrast‐enhanced images show heterogeneous enhancement with areas of necrosis/calcification and possible capsular/lateral wall breach. Features strongly suggest adrenal neoplasm (pheochromocytoma). Right (3D reconstruction). (C) Demonstrates vascular relations of the adrenal mass. The yellow/purple segmentation highlights tumor margins and their relation to renal vessels and inferior vena cava, essential for preoperative surgical planning.

On abdominal imaging, Figure 3A–C demonstrates the adrenal pathology in detail. The coronal CT (Figure 3A) reveals a heterogeneous right suprarenal mass abutting the superior pole of the kidney and extending toward the liver. Contrast‐enhanced imaging (Figure 3B) shows heterogeneous enhancement with internal areas of necrosis and coarse calcifications, along with features suggestive of capsular or lateral wall breach, all strongly favoring the diagnosis of pheochromocytoma. Three‐dimensional reconstruction (Figure 3C) further delineates the tumor margins and highlights its close relationship to the renal vessels and inferior vena cava, providing essential anatomical detail for surgical planning.

## 3. Discussion

Pheochromocytomas are uncommon catecholamine‐secreting tumors that classically present with the triad of headache, palpitations, and sweating in association with paroxysmal or sustained hypertension [7]. However, as demonstrated in this case, they may present atypically, and their protean manifestations have earned them the title of “the great mimic.”

Renal complications in pheochromocytoma are rare but clinically important. Several mechanisms have been described, including prolonged catecholamine‐induced vasoconstriction, leading to renal ischemia, glomerular injury, and secondary tubular damage [8]. Additionally, hypertension and direct toxic effects of catecholamines can cause endothelial injury, proteinuria, and reduced glomerular filtration [9]. In our patient, renal biopsy revealed only mild glomerular changes with significant tubular injury and dystrophic calcifications, consistent with ischemic tubular damage rather than primary glomerulopathy.

Proteinuria in pheochromocytoma is unusual, and reports of nephrotic‐range proteinuria are extremely limited. Previous case studies have documented reversible proteinuria after adrenalectomy, suggesting a functional rather than structural renal cause [10, 11]. Our patient similarly demonstrated full recovery of renal function and resolution of nephrotic‐range proteinuria following surgical excision of the tumor, supporting the hypothesis that catecholamine excess was the primary driver of renal dysfunction. Initial serological abnormalities raised concern for autoimmune glomerulonephritis; however, renal histopathology played a decisive role in excluding immune‐mediated disease and redirecting the diagnostic pathway toward a catecholamine‐secreting tumor.

A further dimension of this case is its diagnostic challenge. Initial serological workup suggested possible autoimmune glomerulonephritis, with positive ANA and low complement levels. However, the renal biopsy showed minimal glomerular changes and predominantly tubular injury, ruling against primary autoimmune disease. This underscores the potential for pheochromocytoma to masquerade as renal or systemic autoimmune pathology, delaying accurate diagnosis. Such diagnostic confusion has been emphasized in earlier reports, which caution clinicians against attributing severe renal dysfunction solely to autoimmune disease when adrenal masses or unexplained adrenergic symptoms coexist [12, 13]. It shows the importance of a complete diagnostic workup, including imaging and biopsy, to reach the most accurate diagnosis.

A further dimension of this case is its diagnostic challenge. Initial serological workup suggested possible autoimmune glomerulonephritis, with positive ANA and low complement levels. However, the renal biopsy showed minimal glomerular changes and predominantly tubular injury, ruling against primary autoimmune disease. This underscores the potential for pheochromocytoma to masquerade as renal or systemic autoimmune pathology, delaying accurate diagnosis. Such diagnostic confusion has been emphasized in earlier reports, which caution clinicians against attributing severe renal dysfunction solely to autoimmune disease when adrenal masses or unexplained adrenergic symptoms coexist [12, 13].

In terms of management, our patient underwent standard preoperative stabilization followed by adrenalectomy, which remains the gold standard treatment for pheochromocytoma. Literature consistently emphasizes the importance of adequate preoperative alpha‐adrenergic blockade to reduce perioperative cardiovascular complications [4, 7]. Riester et al. [14] highlighted that insufficient preoperative preparation is strongly associated with intraoperative hemodynamic instability and postoperative morbidity. In cases complicated by organ dysfunction, such as renal impairment, supportive therapies like dialysis, as used in our patient, have been reported to optimize patients before surgery [11]. Some authors have also described the adjunctive role of calcium channel blockers and beta‐blockers in cases with resistant hypertension or arrhythmias [12, 13]. Our patient’s management strategy involves temporary hemodialysis and careful optimization before definitive surgery, reflecting the curative potential of adrenalectomy when systemic complications are managed proactively.

## 4. Conclusion

This case emphasizes the importance of maintaining a high index of suspicion for pheochromocytoma in patients with unexplained renal dysfunction, especially when accompanied by paroxysmal symptoms or adrenal masses on imaging. Early biochemical screening with plasma or urinary metanephrines and confirmatory imaging are crucial to avoid misdiagnosis.

## Author Contributions

Anum Rizwan designed the case report structure. Sajid Bhatti supervised the case management, confirmed the diagnosis, and critically reviewed the manuscript. Huda Raja finalized manuscript, corresponding author, and performed literature review. Abdullah Nadeem finalized manuscript and performed literature review. Tajammul Waqar assisted in data analysis, contributed to manuscript writing, and provided critical revisions. Sidra German assisted in data analysis, contributed to manuscript writing, and provided critical revisions. Nahid Raufi assisted in data analysis, contributed to manuscript writing, and provided critical revisions. All authors worked combinedly for the management and reporting.

## Funding

The authors received no financial support for this study.

## Disclosure

All authors read and approved the final manuscript.

## Ethics Statement

Institutional ethics approval was waived as per policy for single case report.

## Consent

Written informed consent for publication of this case report and all accompanying images was obtained from the patient’s legal guardian. A copy of the signed consent form is available for review by the editorial office upon request.

## Conflicts of Interest

The authors declare no conflicts of interest.

## Data Availability

Data sharing is not applicable to this article as no datasets were generated or analyzed during the current study.

## References

[1] Lenders J. W. , Duh Q. Y. , and Eisenhofer G. , et al.Pheochromocytoma and Paraganglioma: An Endocrine Society Clinical Practice Guideline, The Journal of Clinical Endocrinology and Metabolism. (2014) 99, no. 6, 1915–1942, 10.1210/jc.2014-1498, 2-s2.0-84902310939. 24893135

[2] Beard C. M. , Sheps S. G. , Kurland L. T. , Carney J. A. , and Lie J. T. , Occurrence of Pheochromocytoma in Rochester, Minnesota, 1950 Through 1979, Mayo Clinic Proceedings. (1983) 58, no. 12, 802–804. 6645626

[3] Sutton M. G. , Sheps S. G. , and Lie J. T. , Prevalence of Clinically Unsuspected Pheochromocytoma: Review of a 50-Year Autopsy Series, Mayo Clinic Proceedings. (1981) 56, no. 6, 354–360, 10.1016/S0025-6196(26)01464-3. 6453259

[4] Neumann H. P. , Young W. F.Jr, and Eng C. , Pheochromocytoma and Paraganglioma, New England Journal of Medicine. (2019) 381, no. 6, 552–565. 31390501 10.1056/NEJMra1806651

[5] Eisenhofer G. , Pacak K. , and Huynh T. T. , et al.Catecholamine Metabolomic and Secretory Phenotypes in Phaeochromocytoma, Endocrine Related Cancer. (2011) 18, no. 1, 97–111, 10.1677/ERC-10-0211, 2-s2.0-79251512979. 21051559 PMC3671349

[6] Bulane C. M. , Marais D. A. , Dlamini Z. , and Somers A. , Clinical Profile and Outcomes of Patients With Pheochromocytoma and Paraganglioma: Audit of Two Tertiary Hospitals in South Africa, The Journal of the Endocrine Society. (2021) 5, no. 8, bvab087.34159287

[7] Maghakyan S. and Aleksanyan A. , Pheochromocytoma Presenting With Paroxysmal Hypertensive Crises in a Previously Healthy Woman: A Case Report, Cureus. (2026) 18, no. 1, 10.7759/cureus.100754, e100754. 41640925 PMC12867173

[8] Prejbisz A. , Lenders J. W. , Eisenhofer G. , and Januszewicz A. , Cardiovascular Manifestations of Phaeochromocytoma, Journal of Hypertension. (2011) 29, no. 11, 2049–2060, 10.1097/HJH.0b013e32834a4ce9, 2-s2.0-80054096098. 21826022

[9] Van der Horst-Schrivers A. N. , Kerstens M. N. , and Wolffenbuttel B. H. , Prevalence of Cardiovascular Disease and Risk Factors in Pheochromocytoma, Netherlands Journal of Medicine. (2006) 64, no. 6, 219–222. 16990692

[10] Tirri R. , Casiere D. , Mattera E. , Guarino G. , Iacono G. , and Federico P. , Sindrome nefrosica e feocromocitoma. Descrizione di un raro caso clinico [The Nephrotic Syndrome and Pheochromocytoma. A Report of a Rare Clinical Case], Clinical Therapeutics. (1994) 145, no. 9, 199–203, Italian. 7813165

[11] Osawa S. , Hosoya T. , Minami T. , Fushimi K. , Yamaguchi K. , and Hishida A. , Pheochromocytoma Associated With Nephrotic Syndrome: A Case Report and Review of the Literature, Clinical Nephrology. (1993) 39, no. 6, 332–336.

[12] Lee M. J. , Kim M. K. , and Ko S. H. , et al.Atypical Manifestations of Pheochromocytoma in Clinical Practice: A Multicenter Retrospective Study in Korea, Endocrine Journal. (2014) 61, no. 7, 625–633.

[13] Manger W. M. and Gifford R. W. , Clinical and Experimental Aspects of Pheochromocytoma, Trends in Endocrinology and Metabolism. (2002) 13, no. 10, 506–511.

[14] Riester A. , Weismann D. , and Quinkler M. , et al.Life-Threatening Events in Patients With Pheochromocytoma, European Journal of Endocrinology. (2015) 173, no. 6, 757–764, 10.1530/EJE-15-0483, 2-s2.0-84947917050. 26346138

